# Syllable Structure Universals and Native Language Interference in Second Language Perception and Production: Positional Asymmetry and Perceptual Links to Accentedness

**DOI:** 10.3389/fpsyg.2015.01801

**Published:** 2015-11-26

**Authors:** Bing Cheng, Yang Zhang

**Affiliations:** ^1^Department of English, School of Foreign Studies, Xi’an Jiaotong UniversityXi’an, China; ^2^Department of Speech-Language-Hearing Sciences, University of Minnesota, MinneapolisMN, USA; ^3^Center for Neurobehavioral Development, University of Minnesota, MinneapolisMN, USA; ^4^Center for Applied and Translational Sensory Science, University of Minnesota, MinneapolisMN, USA

**Keywords:** native language neural commitment, phonetic learning, speech perception, speech production, accentedness, syllable structure, allophonic variations

## Abstract

The present study investigated how syllable structure differences between the first Language (L1) and the second language (L2) affect L2 consonant perception and production at syllable-initial and syllable-final positions. The participants were Mandarin-speaking college students who studied English as a second language. Monosyllabic English words were used in the perception test. Production was recorded from each Chinese subject and rated for accentedness by two native speakers of English. Consistent with previous studies, significant positional asymmetry effects were found across speech sound categories in terms of voicing, place of articulation, and manner of articulation. Furthermore, significant correlations between perception and accentedness ratings were found at the syllable onset position but not for the coda. Many exceptions were also found, which could not be solely accounted for by differences in L1–L2 syllabic structures. The results show a strong effect of language experience at the syllable level, which joins force with acoustic, phonetic, and phonemic properties of individual consonants in influencing positional asymmetry in both domains of L2 segmental perception and production. The complexities and exceptions call for further systematic studies on the interactions between syllable structure universals and native language interference with refined theoretical models to specify the links between perception and production in second language acquisition.

## Introduction

Accentedness in speech refers the degree to which the pronunciation of an utterance sounds deviated from an expected version of its production (Munro et al., 2006). Foreign accent seems to be a natural and unavoidable consequence as adult learners of a second language (L2) are considered to be “phonologically deaf” to the new language (Listerri, 1995). It is well acknowledged that some L2 sounds are much harder to learn than others. In particular, native-like perception and production for the consonants and vowels that either do not occur in the first language (L1) or are phonologically realized differently can be very difficult to achieve for adult L2 learners. A fundamental question in second language acquisition research is to explain the patterns of perceptual difficulties for different speech sound categories in various phonological contexts in connection with production issues in phonetic learning.

There are three general accounts that, respectively, emphasize the roles of the learner characteristics, input properties, and L1 influences. The *Critical Period* account emphasizes age dependency, which postulates genetically guided maturation of domain-specific language learning mechanisms as the reason for the declining abilities to learn L2 (Lenneberg, 1967; Johnson and Newport, 1989). The *Environmental Influence* account shifts the focus to assimilation of external factors by arguing that L2 proficiency variations reflect the different degrees of environmental influences as learners of various ages are inherently at different levels of cognitive, social, and cultural maturation (Snow, 1983; Jia and Aaronson, 2003). The *L1 Transfer/Interference* account highlights the role of structural compatibility, demonstrating that L2 perception/production is directly influenced by the L1 phonological system (Beckman, 1986; Munro and Derwing, 1995; Guion et al., 2000; Eckman et al., 2003; Iverson et al., 2003; Aoyama et al., 2004; Wang et al., 2004; Rogers and Dalby, 2005). Converging evidence suggests that the L1–L2 structural differences may interfere with speech learning at multiple levels, which include the acoustic and phonetic properties of speech sounds, allophonic variations, phonological rules, and syllable structure. The present exploratory study contributes data to the line of L1 transfer/interference research by investigating the role of syllable structure differences between L1 and L2 in L2 segmental perception and production. A corollary question is to investigate the strength of perception-production links as a function of syllabic position and sound category.

A number of theoretical models have been proposed for the L1 Transfer/Interference account at the segmental level. For instance, the Speech Learning Model (SLM; Flege, 1995, 2003, 2007) addresses how adult speakers acquire L2 consonants and vowels based on phonetic similarity to L1. The model predicts that production accuracy is limited by how accurately the L2 sounds are perceived (MacKay et al., 2001). Thus a major determinant of L2 accentedness is the underlying perceptual problem with the L2 phonology. According to the SLM, learners of all ages retain the capacity to align their production of L2 phonetic segments to long-term memory representations for vowels and consonants in the L2. Similarly, the Perceptual Assimilation Model (PAM; Best, 1995; Best and Tyler, 2007) proposes that L2 listeners classify sound contrasts via different routes of assimilation. L2 contrasts are classified as a Two Category (TC), Category Goodness (CG), or Single Category (SC) contrast, which depends on the similarities between the L2 and L1 sounds associated with different degrees of learning difficulties. The model attributes “malleability” to the perceptual systems of adult L2 learners, and predicts that speech discriminability may improve as a function of L2 experience (Best and Strange, 1992).

While the SLM and PAM are based on behavioral studies alone, the Native-Language-Neural-Commitment (NLNC) model adds a neural-level specification and explanation from both developmental and cross-linguistic perspectives (Kuhl, 2004). There are four main theoretical claims in the NLNC model. (a) Early learning produces neural commitment to the abstract phonetic units and their statistical and combinatorial patterns in the native language in the process of establishing prototypical representations of the phonemic inventories and phonological structures, which can be predictive of language skills at word and sentence levels at later ages (Kuhl et al., 2005). For instance, specific brain regions in adulthood become more sensitive to native speech contrasts with more focal and efficient activation in comparison with non-native speakers (Zhang et al., 2005). (b) The effects of NLNC are self-reinforcing and bidirectional – it enhances efficient processing of compatible higher-order linguistic patterns, while at the same time hindering the detection of non-conforming patterns contained in foreign languages, as shown behaviorally (e.g., Iverson et al., 2003) and neurally at the pre-attentive level (e.g., Zhang et al., 2005). (c) Neural commitment is subject to continual shaping and reshaping by experience – Enriched exposure (including high stimulus variability and talker variability, exaggerated speech, and audiovisual training) not only provides enhanced stimulation to the infant brain (e.g., Zhang et al., 2011) but also can induce substantial plasticity in the adult brain for second language learning, producing hemispheric reallocation of resources for enhanced phonetic sensitivity and more efficient linguistic processing (Zhang et al., 2000, 2009). (d) Neural commitment involves the binding of perception and action systems to facilitate speech communication, and this process depends on social/affective learning early in life (Imada et al., 2006; Kuhl, 2007; Stevens and Zhang, 2014). These claims are consistent with the developmental framework that views language acquisition as an adaptive computational process to extract the abstract speech categories and higher-order linguistic structures. Similar views were expressed by other researchers (e.g., Werker and Tees, 2005). Unlike the SLM and PAM, the NLNC model does not specify how different degrees of acoustic, phonetic, or phonological conformity independently or jointly influence L2 learning in childhood or adulthood, which may result in varying outcomes in the cortical and subcortical brain circuits and networks dedicated to L1 and L2 phonological acquisition.

It is noteworthy that the three models for speech learning draw heavily on data from perceptual tests of consonants at the syllable-initial position alone. Apart from what these segmental learning models can explain and predict, suprasegmental differences between L1 and L2 strongly influence L2 phonetic perception and production. The adult L2 perception and production literature indicates the existence of a positional asymmetry that is attributable to differences in L1 and L2 syllable structures (Anderson, 1983; Flege, 1989; Stockman and Pluut, 1999; Broselow and Xu, 2004; Tsukada et al., 2004; Rogers and Dalby, 2005; Cebrian, 2007; Hayes-Harb et al., 2008). For instance, Mandarin Chinese allows no syllable-final consonants other than two nasals (/n/ and /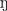/; Duanmu, 2000). Correspondingly, Chinese ESL (English as a Second Language) learner’s perception of stop contrasts was more accurate at the syllable-initial (onset) position than at the syllable-final (coda) position (Flege, 1989), and they also made more production errors in the coda than in the onset (Rogers and Dalby, 2005; Bent et al., 2007). Rogers and Dalby (2005) developed a minimal-pair word list targeting all the English phonemic contrasts that posed difficulty for Mandarin ESL learners and analyzed the intelligibility of Mandarin-accented English (MAE). The MAE segmental misperceptions for native English listeners occurred more frequently at the word-final position than at the word-initial position. The frequent word-final misperceptions included nasals, voiced obstruents, and consonantal clusters. These patterns indicate that Mandarin speakers have more difficulty in accurately producing consonants in the coda than in the onset. Similar positional asymmetry findings were previously reported in other studies, confirming the effect of L1 syllable structure and phonotactics on L2 learning (Eckman, 1981; Flege, 1995).

One straightforward explanation is that the syllable-final difficulties in L2 consonantal perception and production may reflect a general pattern of universal structural asymmetry in favor of the onset in terms of accessibility, stability, and learnability (Ohala, 1996; Carlisle, 2001; Nam et al., 2009). If the positional asymmetry is universally applicable and strong regardless of language experience, one would expect it to occur even in normal L1 learners. Moreover, if the positional asymmetry is a dominant force in L2 phonetic learning, it could potentially overrule the influences of acoustic, phonetic, and phonemic factors and be applicable to all classes of consonants. Given the constraint of syllable structure universals, it also follows that correlations between perception and production would be stronger for consonants in the CV context as opposed to VC. While these three conjectures provide the initial theoretical impetus for the current investigation, our interest is not limited to the verification or falsification of this language-universal structural preference account. Rather, we are intrigued by three basic observations in L2 phonetic learning: (a) positional asymmetry occurs in both domains of perception and production, (b) certain classes of sounds appear to be more affected than others in terms of positional asymmetry, and (c) the strength of correlations between perception and production appears to vary depending on sound category and syllabic position (Cheng and Zhang, 2009). Speech learning theories generally assume that accurate production of many L2 sounds tends to be challenging for adult learners due to their perceptual difficulties, but there has been a lack of studies to examine systematically the extent to which production errors or accentedness are perceptually based as a function of syllabic position and sound category. There is also no theoretical model that readily provides an exact account for the presence/absence (or rather the strength/weakness) of perception-production correlations in L2 phonetic learning.

The relationship between perception and production has been an outstanding issue in speech science (Massaro and Chen, 2008). In L2 acquisition, only moderate correlations have been reported between perception and production (Flege, 2003; Rauber et al., 2005; Cheng and Zhang, 2009). Some studies show either weak or no correlation between L2 segmental perception and production (e.g., Hattori and Iverson, 2010; Levy and Law, 2010; Kartushina and Frauenfelder, 2014). According to the SLM, perception precedes and guides production with perception accuracy imposing an upper limit on production accuracy. Although this contingency relation takes place in a position-sensitive allophonic level as opposed to the abstract phonemic level, it does not necessarily imply correlation between perception and production (Flege, 2003). Consistent with the argument, research findings show that while appropriate perceptual training methods produce sizable improvements in both perception and production, the amount of perceptual gains does not necessarily align with the amount of gains in production accuracy (Rochet, 1995; Bradlow et al., 1997, 1999; Best et al., 2001). The SLM specifically points out the two possible exceptional scenarios to the general rule that production is perceptually based. The first concerns the lack of sufficient input to allow L2 learning in either production or perception. The second applies to a small set of typologically rare L2 phonetic segments that are mastered relatively late even in normal native speakers of that language. If these two exceptional cases are removed, one would generally expect to see a significant correlation between perception and production in L2 learners. While the SLM spells out the exceptions and emphasizes the need to consider allophonic variations in different contexts, it stops short of providing predictions regarding the relative strength of position-dependent perception-production correlations in adult L2 learners for the different classes of speech sounds.

The main purpose of the present study was to investigate positional asymmetry and the influences of Mandarin Chinese syllable structure on the relationship between perception and production of American English consonants. In one previous report, Mandarin speakers’ perceptual gains on a French /b-p/ contrast during perceptual training resulted in improved production (Rochet, 1995). Importantly, the training effects were found to generalize to other stop contrasts in the syllable-initial position but not in the syllable-final position, suggesting that there might be a stronger correlation between perception and production in the syllable-initial position. There are at least two related issues here in terms of L1 transfer/interference. First, as previous studies have shown and argued, production error patterns at the onset and coda positions reflect interference of the native language syllable structure. Second, poorer L2 perception may lead to more accented speech as well as production errors, and the strength of the perception-production relationship could be further influenced by L1 syllable structure. By contrast, an alternative possible explanation is that the positional asymmetry as well as the strength of perception-production links may reflect universal structural preference of CV over VC. In this perspective, native language interference could be a subordinate factor. Although the positional asymmetry effect is attributable to structural differences between L1 (Mandarin Chinese) and L2 (American English) at the syllable level, it remains to be tested whether the effect applies across the board to all classes of L2 consonants that are not permissible in the L1 syllable-final position. It is also equally important to examine whether the positional asymmetry can be found in both native and non-native speakers and whether it applies to the L2 consonants that are allowed in the L1 syllable-final position, both of which would be consistent with the universal structural asymmetry account.

A comparison of Mandarin Chinese (also known as Putonghua in mainland China) and American English phonemes and syllable structures is necessary to help understand the syllable structure differences and the consonant systems of the two languages. There are approximately 400 syllables in Mandarin without tones and 1100 syllables with the tones added (Chao, 1968; Duanmu, 2000). In contrast, English has a much larger inventory of permissible syllables, which exceeds 80,000. At the individual sound level, Mandarin has three nasal sounds as in English. Unlike the English voicing distinction, the bilabial, alveolar, and velar stops in Mandarin are distinguished by the presence or absence of aspiration. In other words, all the Mandarin Chinese stops are voiceless sounds. Similarly, the fricatives and affricates in Chinese also rely on the aspiration distinction instead of voicing. English has more fricatives (9 in total) than Mandarin (5 in total). Except for the glottal /h/, English fricatives carry the voicing distinction for the place of articulations (POAs) of labiodental, interdental, alveolar, and palatoalveolar. There are no Mandarin equivalents for the English interdental fricatives, /θ/ and /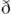/. In contrast, Mandarin has a richer set of affricates, namely, the alveolar /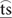/ and /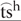/, the retroflex /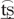/ and /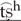/, and the palatoalveolar /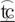/ and /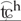/, each pair carrying the aspirated-unaspirated distinction. In English, there is only one pair of palatoalveolar affricates with the voicing distinction (/tʃ/ and /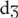/). In the approximant category, Mandarin has liquids and glides like English. But the Mandarin ‘r’ sound is often transcribed as /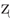/ (a voiced retroflex fricative). The two languages also differ in phonotactic constraints in terms of combinatory and distributional restrictions. While consonants can form clusters in American English words, no consonant clusters are allowed in Mandarin. The inventory of coda structures in English is considerably richer than Chinese. For the current study, we take into account 18 consonants that could appear in both initial and final position of the syllable in English (see **Table 1** for sample words). English also allows all consonants to appear in either syllable-initial or syllable-final position except /h/, /j/ and /w/. In Mandarin Chinese, only two nasal sounds, /n/ and /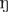/, are permissible in the syllable-final position. In both languages the velar nasal sound /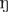/ cannot occur in the word-initial position.

**Table 1:** Sample words used in the study.

	Examples	
	
Target Phoneme	Syllable-initial	Syllable-initial
m, n, 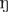 ^∗^	Meat, neat	Seem, seen, sing
b, p, d, t, g, k	Bit, pit, dean, teen, gum, come	Rib, rip, need, neat, dug, duck
f, v	Fine, vine	Leaf, leave
θ, 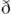	Thin, then	Bath, bathe
s, z	Sip, zip	Loose, lose
ʃ	Sheet	Leash
tʃ, 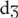	Chin, gin	Rich, ridge
l, 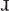	Lead, read	Heal, here


*^∗^/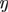/ is not allowed in the onset. /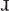/ in the syllable-final position is rhotacized into the r-colored vowel in American English. The data for /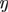/ and /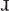/ were not included in the statistical analysis of positional asymmetry.*

In the present study, an overall assessment of consonantal perception and production skills in adult ESL learners in China was attempted by examining effects for all the major individual classes of English consonants in terms of voicing, POA, and manner of articulation (MOA) in the CVC (consonant-vowel-consonant) syllabic context. Testing all the major classes of English consonants was partly motivated by the pedagogical need to identify general patterns and issues in perception and production among Chinese college students for whom English is part of their core curriculum. For comparison purpose, perception data were also collected from adult native speakers of English using the same set of speech stimuli to verify whether native English speakers would show a similar positional asymmetry to the Chinese subjects. The voicing subcategories in this study refer to the two-way distinction of voiced and voiceless consonants. The POA subcategories refer to the six-way distinction of bilabial, labiodental, dental (interdental), alveolar, palatal-alveolar, and velar consonants. The MOA subcategories refer to the five-way distinction of nasal, plosive, fricative, affricate, and approximant (including both liquids and glides) consonants.

There were two specific hypotheses for our study motivated by the three models on L2 phonetic learning and the universal structural asymmetry theory. First, adult Chinese ESL learners would experience more difficulties in perceiving and producing English consonants at the syllable-final position relative to the syllable-initial position across all the voicing, POA, and MOA subcategories due to the limitations of Mandarin Chinese syllabic and phonotactic structures. We did not expect the native speakers of English would have the same perceptual issue although the universal structural asymmetry theory would predict otherwise. Second, the position-dependent difficulties would be reflected in stronger correlations between L2 perception and production at the syllable onset than at the coda.

Considering the syllable structure of Mandarin Chinese and the complexity of the causes for L2 phonetic learning difficulties due to the relative importance of acoustic, phonetic, phonemic properties of the individual speech sounds in each subcategory, we expected to see category-dependent variations in the positional asymmetry phenomenon as well as exceptions to our two general hypotheses at the individual sound level. In particular, as the Chinese language allows /n/-/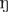/ contrast at the coda position, our expectation, in line with the three models of L2 phonetic learning, was that the Chinese ESL learners would not experience much difficulty in producing or perceiving this syllable-final nasal contrast in English. In other words, the nasal sounds might not show the positional asymmetry. As the POA has more subcategories tied to individual sounds (e.g., the labiodental /f/ and /v/, interdental /θ/ and /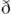/, palatal /ʃ/, palatoalveolar /tʃ/ and /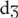/; see **Table 1** for the list of target phonemes) than voicing and the MOA, we might see a constellation of exceptions in the POA subcategories. In accordance with the SLM and PAM, English sounds such as the voiced stops, voiced fricatives/affricates, interdental fricatives, and palatoalveolar affricates that do not exist in Chinese would show different degrees of perception and production difficulties with some of these sounds demonstrating equal difficulties or reduced positional disparity between the syllable onset and coda.

## Materials and Methods

### Participants

The reported study was conducted with approval from the institutional review boards for human subject protection at the two home institutions. Thirty-nine Chinese college students participated in this study. One Chinese female student did not complete all the tests in the study and was dropped in statistical analysis. The Chinese participants were between 20 and 21 years old (29 females and 10 males). All of them were native speakers of Mandarin Chinese. When selecting participants, we excluded students who spoke Mandarin Chinese (also referred as “Putonghua”) with a noticeable accent. Our survey of language background indicated that all our Chinese participants grew up speaking standard Mandarin Chinese from the first year of age. While eight (six females and two males) of the 38 participants were raised in regions where the local dialects (Wu and Min) were very different from Putonghua (Wang, 1991), they all learned the standard Mandarin early on and predominantly spoke Putonghua since early childhood. For cross-language comparison, a group of 12 native American English speakers participated in the perception test subjects (age: 19–22; 7 females and 5 males). All the participants were volunteers under informed consent and recruited after screening for speech, hearing, and language background.

To reduce possible confounds on the perception-production correlations due to insufficient input/learning as specified in the SLM, we were rather restrictive in recruiting the Chinese subjects with respect to their L2 background. The Chinese subjects had received at least 6 years of formal English education in junior and senior high school, in which reading and writing proficiency in English were emphasized rather than conversational skills. None had lived in an English-speaking community before joining the study. All the Chinese participants had passed the National Matriculation English Test in China for college admission, and all except one had passed the national standardized English proficiency test, Test for English Majors, TEM-4 (Zou, 2003; Cheng, 2008), before joining the study. Their average score for TEM-4 was 71.3 out of 100 points with a standard deviation of 7.7, and the average listening comprehension score was 24.8 out of 30 points with a standard deviation of 3.2. The one participant who failed the TEM-4 was also included in our study because this person had a 70% passing grade (21 out of 30) for the listening comprehension section. According to the TEM-4 syllabus, a passing listening grade for TEM-4 indicated that the examinee had a vocabulary knowledge of approximately 6000, and was able to understand speeches or conversations by native English speakers about daily and social life, news broadcasts of BBC and VOA at normal speed, and listening passages comparable to the mini talks in TOEFL (Test of English as a Foreign Language).

### Perception Test

The perception data were collected from both subject groups with a revised Speech Assessment and Training software program (SAT) based on versions used in previous publications (Zhang et al., 2000; Cheng and Zhang, 2009, 2013; Zhang and Cheng, 2011). In the current implementation, isolated word stimuli were used, containing all consonants with Standard American English pronunciation (see **Table 1** for examples and the target phonemes). To control stimulus familiarity, all word candidates were selected from the published vocabulary list required for College English Test (CET Band 4) in China (Zheng and Cheng, 2008). The word stimuli were spoken by four adult native speakers of American English (two males and two females in the age range of 20 ∼ 32), and digitally recorded with Presonus Firebox sound card and Sennheiser ME65 microphone in a sound treated booth (Acoustic Systems, ETS-Lindgren). None of these four native English speakers participated in the perception test. Before the digital recording, the native English speakers were asked to familiarize themselves with all the words on the word list. They were then instructed to read each word three times in isolation with approximately 1 s silence in between. One clear pronunciation from each speaker was then chosen for each word. For the purpose of testing positional asymmetry, words with consonant clusters were not included to avoid complications due to phonotactic constraints for permissible consonant clusters in the onset and coda positions. The sound levels for all the chosen words were normalized to have equal RMS (root mean square) average intensity using the SONY Sound Forge 9 program. Allophonic variations in different vocalic contexts were included for each English consonant.

Before the perception test, the subjects were familiarized with the visual phonetic symbols along with alphabetic letter spelling shown on a computer monitor with target sounds marked in each word. After ensuring that the subjects were confident about recognizing all the symbols for the consonants and verifying their symbol recognition accuracy, each phoneme in the perception test was presented 10 times using different words, respectively, in the syllable onset and coda positions. Listeners were explicitly instructed to pay attention to possible target consonant candidates as prompted on computer screen and make choices by clicking the proper symbol. The listeners were asked to rely on their immediate impression of the consonant in the target onset or coda position irrespective of word candidacy or meaning. The word stimuli were presented at 70 dB SPL in random order. The subjects were tested individually in a sound booth. The selected target responses were automatically registered in a Microsoft ACCESS database in the SAT program and sorted into syllable onset and coda positions for each subject.

### Production Data Collection and Accentedness Rating

Production data were digitally recorded from each Chinese participant, featuring isolated words that contained the contexts for allophonic variations of the target English consonants. The order of perception and production tasks was counterbalanced among the subjects. Each word was presented on computer screen, the subjects were asked to pronounce it in isolation three times with approximately 1 s pause in between. The subjects were instructed to pronounce the words clearly each time. The spoken words were then normalized in RMS intensity and assessed by two native speakers of English, who had formal training in phonetics. The SONY Sound Forge 9 program and a Sennheiser HD280 Pro binaural headphone were used on a workstation installed with the Presonus Firebox sound card for the rating task.

While production accuracy judgment only permits a binary choice, the accentedness rating measure is designed to allow more gradient reflection of the degrees of deviation from the target pattern of speech production as expected of a native speaker. For this reason, the accentedness rating measure was adopted in the present study. The ratings for the target consonants were in the range of 1 (wrong consonant or very strong foreign accent) and 5 (correctly produced English consonant, no foreign accent) following a foreign accent rating scale similar to Munro and Derwing (1995). The raters were instructed to focus on the highlighted target consonant for each word written in a column in a Microsoft Excel spreadsheet. The raters used continuous scores with decimal points to allow finer distinctions in rating. The raters were instructed to adjust the sound volume so that they could hear the words clearly. In cases of uncertainty, they were allowed to listen one more time to help determine the score. The rating scores for each target consonant in each position were based on the overall assessment of the three consecutive pronunciations of each word. The scores were individually entered and saved for further analysis.

### Data Analysis

The raw scores for each subject were sorted, grouped, and averaged for syllable-initial and syllable-final positions based on the subcategories of voicing, POA, and MOA. The data for the glides (/w/ and /j/) and the glottal fricative /h/ were not included as they did not occur in the syllable-final position in English. Statistical analyses were performed using Systat12. For the perception and production scores, repeated-measures ANOVA tests were performed separately to examine the main effects of syllabic position (onset vs. coda), consonant classification (two classes for voicing, six classes for POA, and five classes for MOA), and possible interactions. As the raters used continuous rating scores, inter-rater and intra-rater reliability for the production data was assessed using Pearson’s correlation analysis. *Post hoc* pair-wise *t*-tests were performed for the individual consonant subcategories to examine the effects of syllable structure on perception and production. To examine the overall positional effect on the perception-production links, correlational analysis was first performed on the overall pooled perception and production scores separately for syllable-initial and syllable-final positions. Correlational tests between perception and production as a function of syllabic position were also run for each class of speech sounds. To examine possible dialectal influences, we additionally performed statistical analyses with dialect as a between subjects factor using a multivariate analysis of variance (MANOVA) model. Given the heterogeneity of the dialectal background of the eight subjects who were from regions where the local dialects are very different from Putonghua, we also performed further analysis by removing these eight subjects from our data set to verify the statistical results.

## Results

### Speech Perception Data

**FIGURE 1:**
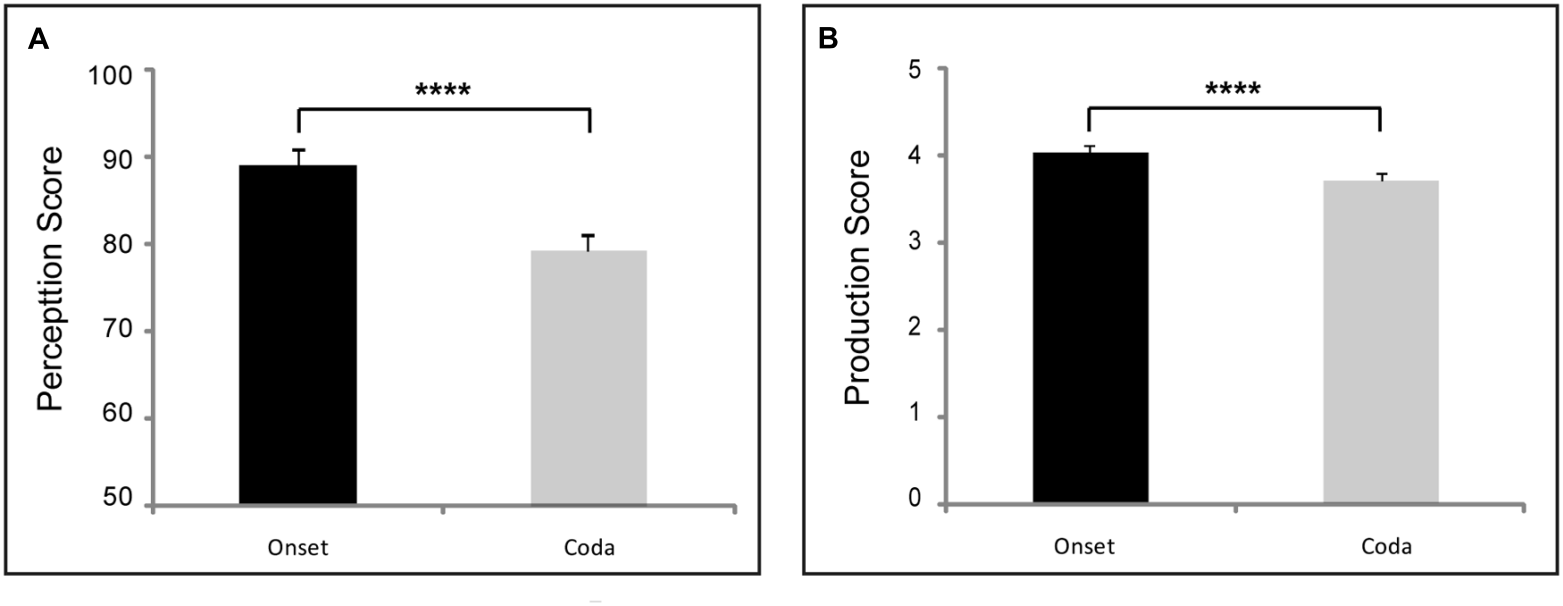
**Positional (syllable onset vs. offset) asymmetry in pooled perception **(A)** and production **(B)** data of all 38 Chinese subjects.** [^∗∗∗∗^ stands for *p* < 0.0001; error bars for a within-subject design are used (Masson and Loftus, 2003)].

**FIGURE 2:**
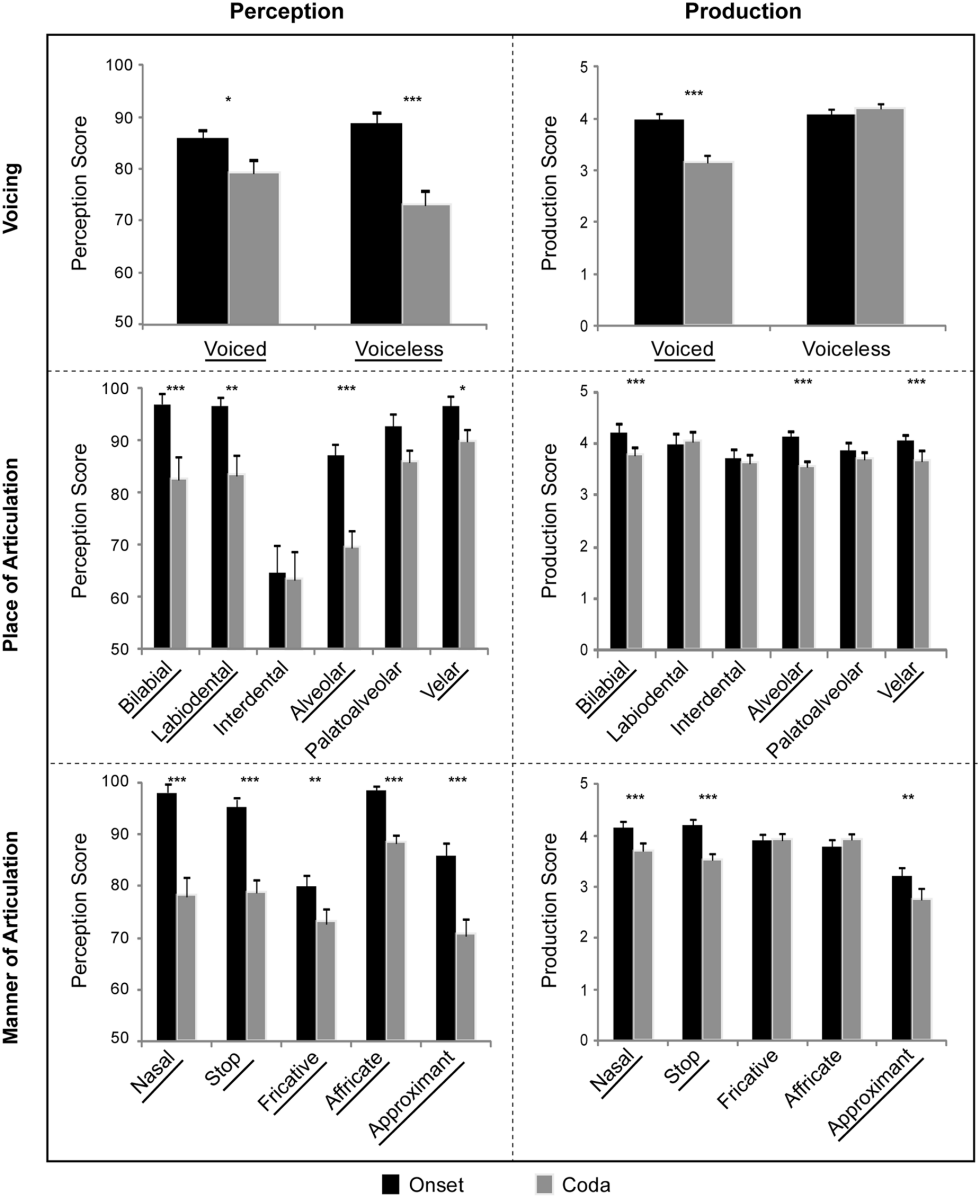
**Perception and production data of all 38 Chinese subjects separated by subcategories of voicing, place of articulation, and manner of articulation.** Each consonant subcategory that showed significant positional asymmetry effect carries the underline mark. (^∗∗∗^*p* < 0.001, ^∗∗^*p* < 0.01, ^∗^*p* < 0.05.)

The pooled perception data and average scores sorted in terms of voicing, POA and MOA categories are shown in the left panels of **Figures 1** and **2** for the Chinese subjects. As expected, the native speakers of English showed ceiling-level perceptual performance for all the consonant categories tested (accuracy range: 95.4–99.2%; standard deviation range: 0.02–0.06). Repeated measures ANOVA results and paired *t*-tests for the grouped consonant categories showed no statistically significant positional asymmetry effects for the monolingual English speakers. As the focus of the study was on L2 perception and production in terms of positional asymmetry, the reported statistics in the remainder of the text would be on the Chinese speakers only. As expected, the Chinese subjects performed significantly better for consonants in syllable-initial position than for those in syllable-final position. There were also exceptions.

In the two voicing subcategories (voiced vs. voiceless), significant effects were found for the main factor of syllabic position [onset versus coda; *F*(1,37) = 49.2, *p* < 0.00001, η^2^ = 0.571] and an interaction between position and voicing [*F*(1,37) = 5.05, *p* < 0.05, η^2^ = 0.120]. There was no main effect of voicing [voiced versus voiceless; *F*(1,37) = 0.82, *p* = 0.4, η^2^ = 0.021]. Further tests of simple main effects showed that significant position effects (onset vs. coda differences) for both the voiced [*F*(1,37) = 6.6, *p* < 0.05, η^2^ = 0.151] and voiceless [*F*(1,37) = 30.2, *p* < 0.00001, η^2^ = 0.449] consonants. A *post hoc* two-tailed paired *t*-test was run to compare the difference scores (onset minus coda) for voiced and voiceless consonants. The result indicated that the voiceless sounds in English had larger onset-coda perceptual difference than the voiced sounds for the Chinese subjects [*t* = 2.248, *p* < 0.05, *df* = 37, Cohen’s *d* = 0.589].

In the six POA subcategories (bilabial, labiodental, interdental, alveolar, palatal-alveolar, and velar), significant effects were observed for the main factors of position [onset versus coda; *F*(1,37) = 43.5, *p* < 0.00001, η^2^ = 0.541], place [*F*(5,185) = 42.0, *p* < 0.00001, η^2^ = 0.532; Greenhouse-Geisser corrected]. There was also an interaction between position and place [*F*(5,185) = 7.1, *p* < 0.001, η^2^ = 0.159; Greenhouse-Geisser corrected]. Simple main effects tests showed significant differences among the POA subcategories at both the onset [*F*(5,185) = 33.9, *p* < 0.00001, η^2^ = 0.478] and coda [*F*(5,185) = 20.0, *p* < 0.00001, η^2^ = 0.351]. Significant position effects were observed for the bilabial [*F*(1,37) = 16.5, *p* < 0.001, η^2^ = 0.308], labiodental [*F*(1,37) = 12.4, *p* < 0.01, η^2^ = 0.251], alveolar [*F*(1,37) = 134.3, *p* < 0.00001, η^2^ = 0.784], and velar [*F*(1,37) = 4.2, *p* < 0.05, η^2^ = 0.101] sounds. *Post hoc* paired *t*-tests (two-tailed; Bonferroni-corrected) were performed for each POA category, which confirmed significant syllabic position effects for the bilabial, labiodental, alveolar and velar sounds: bilabial [*t* = 4.06, *p* < 0.001, *df* = 37, Cohen’s *d* = 0.968], labiodental [*t* = 3.518, *p* < 0.01, *df* = 37, Cohen’s *d* = 0.815], alveolar [*t* = 11.59, *p* < 0.00001, *df* = 37, Cohen’s *d* = 2.38], and velar [*t* = 2.041, *p* < 0.05, *df* = 37, Cohen’s *d* = 0.489]. The interdental and palatoalveolar sounds, which were not part of the Mandarin Chinese phonemic inventory, were the exceptions: interdental [*t* = 0.116, *p* = 0.91, *df* = 37, Cohen’s *d* = 0.024], and palatoalveolar [*t* = 1.886, *p* = 0.07, *df* = 37, Cohen’s *d* = 0.369].

In the five MOA subcategories (nasal, plosive, fricative, affricate, and approximant), the significant effects were as follows: position [onset vs. coda; *F*(1,37) = 194.1, *p* < 0.000001, η^2^ = 0.84], manner [*F*(4,148) = 35.4, *p* < 0.0001, η^2^ = 0.489; Greenhouse-Geisser corrected], interaction of position and manner [*F*(4,148) = 37.8, *p* < 0.0001, η^2^ = 0.505; Greenhouse-Geisser corrected]. Simple main effects tests showed significant differences among the MOA subcategories at both the onset [*F*(4,148) = 13.0, *p* < 0.00001, η^2^ = 0.260] and coda [*F*(4,148) = 48.1, *p* < 0.00001, η^2^ = 0.565]. In addition, significant position effects were observed for all the MOA subcategories, namely, the nasals [*F*(1,37) = 39.3, *p* < 0.00001, η^2^ = 0.515], stops [*F*(1,37) = 50.2, *p* < 0.00001, η^2^ = 0.576], fricatives [*F*(1,37) = 8.7, *p* < 0.01, η^2^ = 0.191], affricates [*F*(1,37) = 42.3, *p* < 0.00001, η^2^ = 0.539], and approximants [*F*(1,37) = 150.3, *p* < 0.00001, η^2^ = 0.803]. *Post hoc* paired *t*-tests (two-tailed; Bonferroni-corrected) confirmed significant syllabic position effects for each MOA subcategory: the nasals [*t* = 6.266, *p* < 0.00001, *df* = 37, Cohen’s *d* = 1.356], stops [*t* = 7.085, *p* < 0.00001, *df* = 37, Cohen’s *d* = 1.55], fricatives [*t* = 2.953, *p* < 0.01, *df* = 37, Cohen’s *d* = 0.579], affricates [*t* = 3.212, *p* < 0.001, *df* = 37, Cohen’s *d* = 0.916], and approximants [*t* = 12.26, *p* < 0.00001, *df* = 37, Cohen’s *d* = 2.398].

### Speech Production Data

The accentedness ratings for the Chinese subjects in terms of voicing, POA and MOA subcategories are summarized in the right panels of **Figures 1** and **2.** The inter-rater correlation was significant [Pearson’s *r* = 0.73, *p* < 0.001]. Intra-rater correlations were further performed by half-splitting the individual ratings for the same consonants in each subject, and the results showed a high level of consistency for both raters [Pearson’s *r* = 0.86 for rater 1, Pearson’s *r* = 0.89 for rater 2; *p* < 0.0001 for both raters]. The scoring data indicated that the two native English speakers with training in phonetics were highly consistent in their accentedness judgment. The two raters’ scores were thus averaged for each sound tested for each subject. Overall, the subjects’ consonantal production was rated significantly better (i.e., less accent) for syllable-initial position than syllable-final position. There were also some exceptions in individual subcategories as reported below.

In the two voicing subcategories (voiced vs. voiceless), the significant effects were as follows: syllabic position [onset versus coda; *F*(1,37) = 37.5, *p* < 0.0001, η^2^ = 0.764], voicing [voiced versus voiceless; *F*(1,37) = 119.5, *p* < 0.00001, η^2^ = 0.503], interaction of position and voicing [*F*(1,37) = 70.2, *p* < 0.00001, η^2^ = 0.655]. In tests of simple main effects, a significant position effect (onset vs. coda differences) was observed for the voiced consonants [*F*(1,37) = 61.8, *p* < 0.00001, η^2^ = 0.626], and significant differences between the voiced and voiceless sounds were found at the coda [*F*(1,37) = 125.4, *p* < 0.00001, η^2^ = 0.228]. *Post hoc* paired *t*-tests showed a significant position effect for accentedness in voiced consonants [*t* = 7.863, *p* < 0.00001, *df* = 37, Cohen’s *d* = 1.819] but not in the voiceless consonants [*t* = -1.44, *p* = 0.136, *df* = 37, Cohen’s *d* = -0.188].

In the six POA subcategories (bilabial, labiodental, interdental, alveolar, palatal-alveolar, and velar), significant effects in accentedness were found for the main factors of position [onset versus coda; *F*(1,37) = 34.9, *p* < 0.00001, η^2^ = 0.485] and place [*F*(5,185) = 10.2, *p* < 0.0001, η^2^ = 0.217; Greenhouse-Geisser corrected]. There was also an interaction between position and place [*F*(5,185) = 10.4, *p* < 0.0001, η^2^ = 0.22; Greenhouse-Geisser corrected]. Simple main effects tests showed significant differences among the POA subcategories at both the onset [*F*(5,185) = 6.4, *p* < 0.001, η^2^ = 0.148] and coda [*F*(5,185) = 12.6, *p* < 0.0001, η^2^ = 0.253]. Significant position effects were observed for the bilabial [*F*(1,37) = 19.8, *p* < 0.0001, η^2^ = 0.348], interdental [*F*(1,37) = 8.8, *p* < 0.01, η^2^ = 0.191], alveolar [*F*(1,37) = 47.8, *p* < 0.00001, η^2^ = 0.563], and velar [*F*(1,37) = 45.7, *p* < 0.00001, η^2^ = 0.553] sounds. *Post hoc* paired *t*-tests were performed for each POA subcategory, which confirmed positional asymmetry in the same four POA subcategories: bilabial [*t* = 4.447, *p* < 0.0001, *df* = 37, Cohen’s *d* = 0.848], interdental [*t* = 2.959, *p* < 0.01, *df* = 37, Cohen’s *d* = 0.463], alveolar [*t* = 6.911, *p* < 0.00001, *df* = 37, Cohen’s *d* = 1.416], and velar [*t* = 6.761, *p* < 0.00001, *df* = 37, Cohen’s *d* = 1.593]. The two exceptions were labiodental [*t* = -0.98, *p* = 0.3, *df* = 37, Cohen’s *d* = -0.221], and palatoalveolar [*t* = 0.513, *p* = 0.6, *df* = 37, Cohen’s *d* = 0.099].

In the five MOA subcategories (nasal, stop, fricative, affricate, and approximant), the significant effects in accentedness were as follows: position [onset vs. coda; *F*(1,37) = 64.9, *p* < 0.000001, η^2^ = 0.637], manner [*F*(4,148) = 50.1, *p* < 0.00001, η^2^ = 0.575; Greenhouse-Geisser corrected], interaction of position and manner [*F*(4,148) = 5.9, *p* < 0.01, η^2^ = 0.138; Greenhouse-Geisser corrected]. Simple main effects tests showed significant differences among the MOA subcategories at both the onset [*F*(4,148) = 39.1, *p* < 0.00001, η^2^ = 0.514] and coda [*F*(4,148) = 12.6, *p* < 0.0001, η^2^ = 0.431]. In addition, significant position effects were observed for the nasals [*F*(1,37) = 21.8, *p* < 0.0001, η^2^ = 0.371], stops [*F*(1,37) = 80.5, *p* < 0.00001, η^2^ = 0.685], and approximants [*F*(1,37) = 9.4, *p* < 0.01, η^2^ = 0.202]. *Post hoc* paired *t*-tests confirmed significant syllabic position effects for the nasals [*t* = 4.671, *p* < 0.0001, *df* = 37, Cohen’s *d* = 0.986], stops [*t* = 8.97, *p* < 0.00001, *df* = 37, Cohen’s *d* = 1.984], and approximants [*t* = 3.06, *p* < 0.01, *df* = 37, Cohen’s *d* = 0.591]. There were two exceptions; namely, fricatives [*t* = 1.305, *p* = 0.2, *df* = 37, Cohen’s *d* = 0.235], and affricates [*t* = 1.374, *p* = 0.1, *df* = 37, Cohen’s *d* = 0.241].

### Correlations between Perception and Production

**FIGURE 3:**
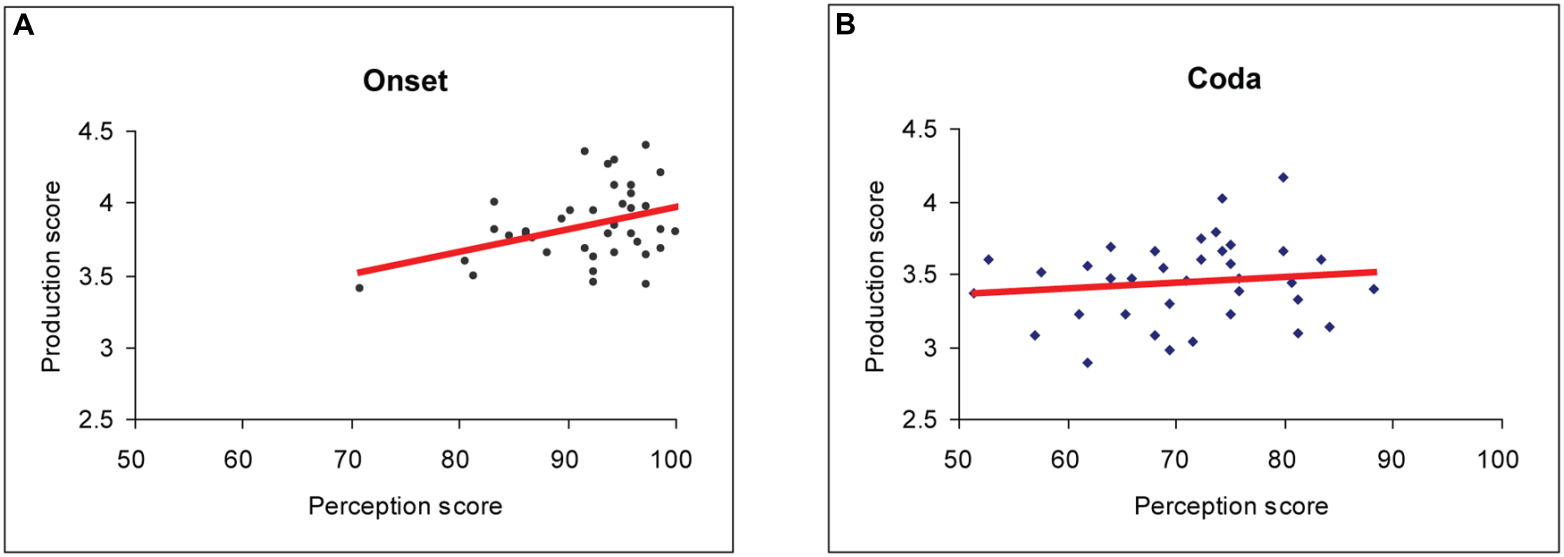
**Pearson’s correlation analysis results for pooled perception and production data from all 38 Chinese subjects.** **(A)** Significant correlation was found at the syllable-initial position (Pearson’s *r* = 0.37; *p* < 0.05; Bonferroni-corrected). **(B)** No significant correlation was found at the syllable-final position.

Pearson’s correlation results for the grand mean scores of the Chinese subjects showed significant positive links between perception and production in the syllable-initial position [Pearson’s *r* = 0.4; *p* < 0.05] (**Figure 3A**). In contrast, no significant correlational effect was observed between perception and production for the syllable-final position [Pearson’s *r* = 0.12; *p* = 0.5] (**Figure 3B**).

Detailed correlational results for each of the voicing, POA, and MOA subcategories were summarized in **Table 2.** For all the voicing and MOA subcategories, significant positive correlations were observed only in the onset but not in the coda. It is noteworthy that contrary to our prediction, the nasal sound category also showed positional asymmetry in perception and production as well as in the correlational analysis. Exceptions were also found in the POA subcategories. In particular, the labiodental and interdental sounds did not show significant correlations in the onset; and contrary to our hypothesis, the palatoalveolar sounds showed significant correlation in the coda.

**Table 2:** Pearson’s correlation analysis results for the perception and production scores in the onset and coda positions from all 38 Chinese subjects, respectively, for the voicing, place of Articulation (POA), and manner of articulation (MOA) subcategories.

Perception-Production Correlation		Onset	Coda
**Voicing**
	Voiced	*r = 0.325*, *p = 0.046^∗^*	*r* = 0.274, *p* = 0.096
	Voiceless	*r = 0.439, p = 0.006^∗∗^*	*r* = 0.072, *p* = 0.668

**POA**
	Bilabial	*r = 0.322*, *p = 0.048^∗^*	*r* = 0.283, *p* = 0.085
	Labiodental	*r = 0.202*, *p = 0.076*	*r* = 0.117, *p* = 0.483
	Interdental	*r = 0.243*, *p = 0.142*	*r* = -0.029, *p* = 0.861
	Alveolar	*r = 0.492*, *p = 0.002^∗∗^*	*r* = -0.093, *p* = 0.577
	Palatoalveolar	*r = 0.366*, *p = 0.024^∗^*	*r = 0.322*, *p = 0.049^∗^*
	Velar	*r = 0.359*, *p = 0.027^∗^*	*r* = 0.309, *p* = 0.059

**MOA**
	Nasal	*r = 0.482*, *p = 0.002^∗∗^*	*r* = -0.093, *p* = 0.577
	Stop	*r = 0.346*, *p = 0.033^∗^*	*r* = 0.205, *p* = 0.216
	Fricative	*r = 0.362*, *p = 0.026^∗^*	*r* = 0.068, *p* = 0.685
	Affricate	*r = 0.328*, *p = 0.045^∗^*	*r* = 0.283, *p* = 0.086
	Approximant	*r = 0.406*, *p = 0.012^∗^*	*r* = 0.179, *p* = 0.284


*Significant correlations are bold-underlined. (^∗∗^p < 0.01; ^∗^p < 0.05.)*

### Statistical Results Concerning Dialectal Influences

Multivariate analysis of variance analyses showed that none of the main effects or interaction effects concerning the dialect factor reached the level of statistical significance (*p* < 0.05) in perception or production (see Tables S1 and S2 in Supplementary Material for details). In the production data for MOA, the main effect of dialect was at the very edge of significance [*F*(1,36) = 4.104, *p* = 0.050]. In addition, in the production data for POA, there was one interaction effect (syllabic position ^∗^ dialect) approaching the level of significance [*F*(1,36) = 3.717, *p* = 0.062].

Further analysis by removing the eight subjects who grew up in the Wu and Min dialect regions essentially replicated the results reported above (see **Supplementary Tables S3**–**S5**). The perception data did not appear to be much affected by removing the eight subjects (**Supplementary Table S3**). In the production data (**Supplementary Table S4**), however, there was a significant position effect for the interdentals [*F*(1,29) = 4.242, *p* < 0.05], which was not observed in the statistical results with all the 38 subjects (**Figure 2**). In the correlational analysis (**Supplementary Table S5**), the MOA affricate subcategory failed to reach the level of significance [*r* = 0.357, *p* = 0.053, *df* = 29] at the syllable onset, but it was a very close miss.

## Discussion

### Positional Asymmetry in L2 Perception

The perception data in terms of voicing, POA, and MOA showed a strong effect of position on perceptual performance at the segmental level in the ESL learners but not in the native English speakers. Overall, the Mandarin Chinese speakers performed significantly better in consonantal perception of English phonemes in the syllable onset position as compared with the coda position for all the subcategories in terms of voicing and MOAs. There were several exceptions in terms of POA subcategories in the perception data. These results are consistent with the positional asymmetry effect reported earlier on stop consonants (Flege, 1989), showing that syllable structure can have a significant impact on phonetic perception (Eckman, 1991; Carlisle, 2001). The ceiling-level performance observed for the control group reflected the relative ease of the perception task for native English speakers, which was aided by the use of a limited number of common words in the CVC context and clearly articulated exemplars as stimuli.

The structural difference at the phonemic and syllabic levels between L1 (Chinese) and L2 (English) jointly showed a direct impact on the perceptual accuracy of individual L2 consonants. Interestingly, we observed a lack of balance among the subcategories of each consonantal classification. The main effects and significant interaction of position and voicing indicate that the Chinese ESL learners’ lack of integration of acoustic cues in the vocalic context for perceiving English stop sounds not only depends on syllabic position but also differs between the voiced and voiceless sounds. For instance, syllabic position showed a stronger effect on perceptual accuracy of voiceless consonants than voiced consonants. This result is not unexpected, suggesting that both syllable structure universals and language-specific factors are at play here (Carlisle, 2001). As the Chinese stops, fricatives, and affricates are voiceless except for /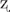/ (a voiced retroflex fricative), the difference in the voiceless vs. voiced sounds could be due to disparity in transferring L1 experience with the voicing distinction to L2 learning. Due to the highly restrictive syllable structure of Mandarin, Mandarin speakers would have more perception and production experience with voiceless consonants in the onset position than voiced consonants, which could partly explain why their perception for the voiceless sounds showed larger onset-coda difference than the voiced sounds. Previous studies showed that Chinese ESL learners had difficulty in the use of vowel lengthening for word-final voiced stops in both perception and production (Flege et al., 1992; Bent et al., 2008).

The POA perception data suggest that L2 phonetic learning difficulties exist at multiple levels, among which structural differences at the syllable level may become a subordinate factor for certain sounds. In particular, the interdental and palatoalveolar sounds did not show a significant effect of syllabic position. As the palatoalveolar sounds happen to be fricatives that are relatively long in duration, the positional asymmetry effect could be partly offset by the presence of long-duration acoustic cues from the preceding vowels that may aid the perception of syllable-final fricative/affricate sounds (Dorman et al., 1980; Jongman et al., 2000; Broersma, 2010). It was previously demonstrated that unlike American English listeners, Mandarin-speaking subjects perceptually weighted the frication noise more heavily than formant transitions and were particularly sensitive to fricative contrasts in the VC context which was phonotactically illegal in their native language (Chiu and Babel, 2010). In agreement with the PAM (Best, 1995), the L1–L2 phonemic inventory differences may also play a major role here. The interdental and palatoalveolar consonants in English do not occur in Mandarin Chinese. Moreover, the interdental fricatives, which were known to be the weakest in terms of acoustic energy in all English consonants, showed the lowest perceptual accuracy. These factors could jointly explain why the fricatives showed the lowest perceptual accuracy among the subcategories of manner of articulation. Further analysis of misperceptions indicated that the interdental sounds were frequently identified as alveolar sounds. This is not surprising as the phonetic learning models, SLM, PAM, and NLNC, would all predict perceptual difficulty with the interdental fricatives for Chinese ESL learners. But the existing models are largely based on primary data of the CV structure, which cannot fully predict or explain the interaction effects as reflected in the presence or absence of the positional asymmetry for particular classes of sounds in L2 perception. For instance, although the Chinese language allows syllable-final nasal contrasts, the Chinese subjects’ perception of English nasals showed a significant positional asymmetry, which seems to support the universal structural preference account (See further discussions below).

### Positional Asymmetry in the Production Data

Consistent with the syllable structure universals theory (Carlisle, 2001), the Mandarin Chinese speakers showed significantly better scores (i.e., lower accentedness) for the production of English consonants in the syllable-initial position than in the syllable-final position. The positional asymmetry echoes previous intelligibility data of Mandarin speakers’ production of English (Rogers and Dalby, 2005; Bent et al., 2007), indicating that Chinese speakers are more likely to produce not only speech errors but also stronger degrees of accentedness in the syllable-final position than in the syllable-initial position.

The significant interaction effects between position and consonant categories indicated that there were differences in the production of various consonantal classes. We observed some discrepancies between perception and production. First, the positional asymmetry effect in terms of voicing was found only for the voiced consonants in production. The overall production of voiceless English consonants by Mandarin Chinese speakers was rated to be more native-like than the voiced ones, reflecting the fact the most of the Chinese consonants are voiceless sounds. Rogers and Dalby (2005) reported a similar production error pattern for word-final voiced obstruents. Second, there was no positional asymmetry effect in the production of labiodentals, interdentals and palatoalveolar sounds. These sounds except for the labiodental /f/ do not exist in Chinese and are typologically rare and even hard for the normal L1 learners, which would fall into the exceptional cases as described in the SLM. Third, the MOA subcategories showed lack of the positional effect for fricatives and affricates. Here syllable structure universals may become subordinate in comparison with the native language interferences and acoustic/articulatory efforts needed for producing these sounds: first, Mandarin Chinese has a richer set of palatoalveolar fricatives and affricates than English, and second, the English interdental and palatoaveolar fricatives and affricates are not in the Chinese phonemic inventory (Ladefoged and Wu, 1984; Duanmu, 2000). As in the case of fricative perception, the amount of duration and effort needed for articulating these fricatives may also play an important role (See Keating et al., 1999 for a discussion about the lack of positional asymmetry in fricative articulation).

One theoretical challenge with the current data is to explain why L1 syllabic structure does not appear to influence L2 production as much as it does in the perceptual domain. Part of the answer may lie in the different scales of measurement we adopted for our study. The perceptual data used a percentage scale of 0–100 whereas the production data were in a much reduced range of 1–5 with no absolute zero involved. Another factor to consider is the relative strength of links between perception and production. The correlation analysis results indicate that there was hardly any significant correlation between perception and production at the syllable-final position. In the present design, the Chinese subjects were instructed to produce the words in isolation, which could have led to increased conscious efforts to articulate each sound in a word regardless of the position. As discussed earlier, phonemic inventory differences and allophonic variations likely play an important role here, too. For example, the English interdental fricatives were the most difficult for the subjects as Mandarin Chinese does not use the interdental POA. The interdentals were so difficult for the Mandarin ESL learners that syllabic position did not seem to matter - their performance for the interdentals was equally poor in the onset and coda for both perception and production. There are also other factors to be taken into account. For instance, successful L2 perceptual training and the perception-induced improvement in production are shown to be dependent on the variability of the input that require multiple talkers and multiple phonetic contexts (Pisoni and Lively, 1995; Zhang and Cheng, 2011). Corpus analysis shows that syllabic constituents in onset and coda positions can be highly predictable as nearly 75% of coda consonants in English are alveolars (Greenberg, 2005). There could be insufficient input/learning for some of the non-alveolar sounds at the syllable-final position, which would then fall into the first exceptional scenario as specified by the SLM. As we could not retrospectively collect data and analyze the input or prior perception/production training that our participants (English majors in a Chinese college) received, our statements about the input quality and quantity remain purely speculative here. Future phonetic training studies can be designed to control and characterize L2 input in terms of combinatory and distributional probabilities in determining the amount of exposure to syllable-initial and syllable-final consonants in different phonetic contexts, which may help explain some of the disparities in learning outcomes that we observed between the consonantal classes.

### Correlations between Perception and Production and Exceptional Cases

Despite the existence of large variances, overall perception and production scores were positively correlated in the syllable-initial position but not in the syllable-final position, which appear to conform to the universal syllable structure pattern in favor of CV over VC. Our data are consistent with previous studies that show a lack of direct correspondence between L2 speech perception and production at the syllable-final position. For instance, Flege (1989) reported that the adult Chinese subjects could identify the final stops in words like ‘beat’ and ‘bead,’ but they pronounced these sounds poorly. In our study, the rating scores of Chinese ESL learners’ production on /t/ and /d/ in the initial position of a word were also better than those in the final position.

As predicted, we saw a constellation of exceptions in the individual POA subcategories. Specifically, significant positive correlations between perception and production were found for palatoalveolar sounds in both onset and coda positions. No significant correlation was found for labiodentals and interdentals in either onset or coda. These exceptions cannot be solely explained with either syllable structure universals or the L1 syllable structure interference account. Given the range of scores, the existence of these exceptions (**Figure 2** and **Table 2**; also see supplementary data) is not due to a lack of variance in either perception or production measures. One technical issue concerning the investigation of the perception-production relationship lies in the lack of commensurability in methodology in terms of experimental protocols and measures.

Dissociation of speech production from perception is well acknowledged in the brain research literature. In studies with brain-lesioned patients, speech perception, and production can be selectively impaired without apparent damage on the other (Praamstra et al., 1991; Dronkers, 1996), which applies to not only to speech but vocal skills in general (including singing; see Hutchins and Moreno, 2013 for a discussion on the Dual Linked Representation model.). Brain imaging studies indicate that white matter morphometric measures and diffusion tensor imaging measures in the left and right auditory cortex as well as other language-related regions (such as the left inferior parietal cortices, the left and right insula) show a dissociation between perception and production in learning foreign speech sounds (Golestani and Pallier, 2007; Golestani et al., 2007).

Consistent with the brain research findings, behavioral data also indicate that L2 perceptual learning is not necessarily paralleled with equivalent gains in the production domain or vice versa. While perception-training-induced improvement in production can be sustained in the long term, it does not necessarily show alignment of proportional gains in the two domains (Bradlow et al., 1997). A number of studies have reported that L2 production may surpass perception (Goto, 1971; Sheldon and Strange, 1982; Flege and Eefting, 1987; Underbakke, 1993; Bohn and Flege, 1996; Beach et al., 2001; Kluge et al., 2007; de Jong et al., 2009a,b; Hattori and Iverson, 2009), which is not in line with predictions of existing theories or models including SLM. The speech learning/acquisition trajectory is most likely non-linear (Zhang and Wang, 2007). Perceptual skills may improve faster than productive skills or vice versa, which varies in individuals that differ in L2 proficiency, age of acquisition, and length of exposure or residence. For instance, it is possible that production might precede perception as a result of production training with explicit explanations of the target articulatory gestures and movements and rigorous exercises with feedback. In this regard, it can be technically difficult to assess measurements and the effects of learning in the two domains in a balanced way using one time measure from a relatively homogenous subject sample.

### Positional Asymmetry in the Special Case of Nasal Sounds

Although the nasal contrast, /n/ and /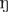/, is permitted in the syllable coda in Mandarin Chinese, there were strong positional effects in both perception and production for the /n/ sound. This pattern is contradictory to our prediction. Moreover, consistent with previous findings (Rogers and Dalby, 2005), we found that the coda /n/ and /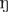/ sounds in English were a difficult contrast for Chinese ESL learners. The current data showed that the English /n/ and /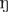/ sounds in coda were not consistently assimilated into the Chinese /n-/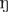/ contrast in either perception or production. Similar results were previously reported in adult Japanese ESL learners, who also showed considerable difficulty in distinguishing /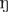/ from /n/ syllable-finally despite the fact that the Japanese language allows nasal coda (Aoyama, 2003).

Acoustic analysis of Chinese words and English loanwords suggests that Chinese coda nasals could be treated as part of the syllable nucleus rather than a consonantal coda (Chen, 2000; Hsieh et al., 2009; see Duanmu, 2000 for a different theorectial view). In this perspective of syllabification, the positional asymmetry effects in perception, production, and the perception-production correlation for the nasal sounds are consistent with the overall patterns across the consonants.

### Positional Asymmetry: A Universal Phonological Constraint?

The perception and production data in this study indicate that in addition to acoustic, phonetic, and phonemic similarities/differences in the set of speech sounds between L1 and L2, there is a strong influence of the L1 syllabic structure in L2 phonetic learning. Could the observed positional asymmetry simply be a reflection of language-universal preference of the CV structure (Tarone, 1978; Singh, 1985; Beckman, 1998; MacNeilage, 1998; Lin, 2003), which is independent of possible interference from the L1 syllable structure? For instance, Golestani et al. (2007) found that when English and Japanese listeners were asked to identify POA for the English consonants, both subject groups performed equally well in the CV context and both had substantially more errors in the VC context. Other studies have reported similar results in adults (Redford and Diehl, 1999; Kochetov, 2004) as well as in infants (Jusczyk et al., 1999; Zamuner, 2006). In production models, consonants in the CV structure are considered to have an advantage over those in VC in terms of accessibility, stability, and learnability (Nam et al., 2009). It is generally agreed that consonants in the syllable onset tend to be produced with greater articulatory effort and precision and longer duration than those in coda (Greenberg, 2005). According to the Positional Faithfulness Theory (Beckman, 1998), the onset-coda differences may reflect a subset of phonological asymmetries and typological optimization within and across languages due to universal positional faithfulness constraints that are driven by perceptual or psycholinguistic prominence/salience in favor of the onset position being the privileged and perceptually strong position. Furthermore, auditory neurophysiology data from animals and humans also support the dominance of onset response over the offset response (Greenberg, 2005).

However, the lack of the positional asymmetry in the perception data from the native speakers of English did not support a straight language-universal interpretation. Phonological theories arguing for the language-universal preference of CV would have difficulty in explaining the exceptional cases as discussed above in both perception and production domains without considering the acoustic, perceptual, articulatory, and co-articulatory details of each sound category in the syllable onset and coda positions and how the interphonology of L1 and L2 accommodates the similarities and differences at the syllable level. We acknowledge that our selection of CVC word stimuli that were clearly articulated in both syllable-initial and syllable-final positions without using a carrier phrase could have increased the predictability of target sounds and eliminated some of the onset-coda articulatory and acoustic differences. For instance, voiced English fricatives in natural connected speech tend to be devoiced in coda positions, but it was not the case in our exemplar stimuli produced in isolation. Developmental data demonstrate that when the onset and coda consonants are clearly articulated, there may be no inherent asymmetry in perceptibility or discriminability for CV and VC syllables for an infant listener (Jusczyk, 1977; Nazzi and Bertoncini, 2009; Swingley, 2009). When careful acoustic manipulation is implemented and experiential factors are controlled, psychoacoustic, and neurophysiological studies suggest that offset coding and the detection of consonants in coda position can be as effective and precise as onset coding (e.g., Pols and Schouten, 1978; Qin et al., 2007; Baltzell and Billings, 2014).

### Native Language Interference: Dialectal Influences

As native speakers of Chinese may represent geographically and linguistically diverse regions with distinct local dialects, it is important to control for dialectal influences in speech research studies. We selected our subject sample by excluding those who spoke Putonghua with an accent. But our sample included eight subjects who are early bilingual speakers of both Putonghua and local dialects that are very different from Putonghua. Statistical tests that specifically examined and removed the dialectal (or rather bidialectal) influences provided verification of the perceptual test results (see **Figure 2** and Table S3 in Supplementary Material). In the production data, there were some minor differences involving the dialect factor that approached the level of statistical significance, which also slightly affected the perception-production correlational test results when those eight subjects were removed in the analysis (see **Table 2** vs. Table S5 in Supplementary Material). As our subject sample did not include those who acquired Mandarin Chinese late and spoke Mandarin Chinese with a noticeable local accent, further studies can probe how dialectal influences (in particular, early vs. late Chinese bidialectalism) factor into the effects of native language interferences in perception and production of the second language.

### Limitations and Alternative Explanations

One methodological limitation of the current study is the use of natural speech stimuli in a CVC context instead of non-sense syllables in the CV and VC formats. Speech research has well established that natural speech contains many redundant cues to segment identity. Both internal cues (heard during the sound) and external cues (heard on the neighboring sounds/syllables) contribute important perceptual information, and language experience may fundamentally change perceptual weighting of such cues (e.g., Cooper et al., 1952; Polka and Strange, 1985; Keating et al., 1999; Zhang et al., 2009). An alternative possible explanation is that the positional effects reflect asymmetry in learning allophonic variations for the CV and VC contexts. However, in the present design, the consonant clusters were not included, and the vocalic influences on segmental identities were not systematically controlled and examined. A number of studies have shown that native speakers of English rely on different acoustic cues and the integration of adjacent consonant and vowel for perceiving the same consonant at syllable-initial, intervocalic, and syllable-final positions (Hillenbrand et al., 1984; Gordon, 1989; Samuel, 1989; Whalen, 1989; Jongman et al., 1992; Pickett et al., 1995; Decoene, 1997; Sinnott et al., 1998; Kochetov, 2004; Nittrouer, 2004). Presumably, the L2 learners would also need to acquire the same set of detailed phonetic knowledge for all the allophonic variations (including consonant cluster contexts) in order to reach native-like performance in perception and production.

A second limitation of the current study is that the results were obtained from English major students in one of the top universities with a highly selective admission rate in China. As perception and production may proceed at different time courses in L2 acquisition, it would be necessary to apply well-defined subject selection criteria for a proper investigation. While efforts were made to include a normally distributed sample size in terms of L2 ability using the standardized TEM-4 scores, the Mandarin-speaking ESL population would be expected to exhibit a much wider range of English proficiency. Further work would be needed to examine whether similar patterns of positional asymmetry in perception and production at the syllable level can be obtained from Mandarin-speaking subjects with lower and higher English proficiency levels as assessed by CET and TEM tests (Zou, 2003; Cheng, 2008).

A third limitation is that the reported results were pooled across consonants for the major classes of voicing, POA, and manner of articulation. While this allows us to see the general patterns of the major consonantal classes, refined studies at the individual sound level are needed to allow a detailed examination of the acoustic, phonetic, and phonological factors at play in the syllable-initial, intervocalic, and syllable-final positions. We currently do not have an exact model for explaining all the exceptions we reported. Existing theories and models including SLM, PAM, NLNC, the Automatic Selective Perception model (Strange, 2011), and the recently revised Second Language Linguistic Perception model (L2LP; Van Leussen and Escudero, 2015) tend to focus on the perceptual side. While it makes sense to argue following the seminal work by Polivanov and Trubetzkoi that foreign accent originates from “the use of language-specific perceptual strategies that are entrenched in the learner” (Escudero, 2007), it is hard to make precise predictions regarding the dissociation patterns between perception and production for the current project of investigation that covers all consonants in the VOT, POA, and MOA classes with respect to the positional asymmetry phenomenon. As such, our study remains rather descriptive and exploratory, which would require more refined research on the individual cases of exceptions to have a better understanding the interactions among the acoustic, phonetic, phonological, and semantic factors in the learning process in both domains of perception and production. In this regard, studying positional asymmetry and the relationship between perception and production in second language acquisition may also benefit from studies of the same nature in the domain of first language acquisition to have a better understanding of the physiological, articulatory, and perceptual factors as well as language-universal and language-specific properties in phonetic learning (e.g., Lin and Demuth, 2015).

### Implications for ESL Education and Future Studies

In practice, ESL teachers are advised that articulatory training would be ineffective unless the problem of perception is overcome (Ur, 1996). This pedagogical practice is supported by evidence from many studies that accurate perception is a prerequisite for good production (Rochet, 1995). However, the current results suggest that only moderate links exist between perception and production and that the perception-production correlation is highly position-dependent at the syllable level. It is important that ESL programs pay special attention to the syllable structure differences between L1 and L2 and the patterns of allophonic variations in both languages to enhance L2 phonetic learning in both perception and production domains. From a pedagogical perspective, the unbalanced relationship between perception and production requires further specification for the role of talker variability in influencing the learning of different classes of speech sounds including both consonants and vowels in different syllabic contexts.

The segmental phonetic learning models such as SLM, PAM, and NLNC all posit that the perceived relationships between L1 and L2 categories play an important role in how accurately L2 segments are perceived or produced. But these models have not fully taken into account production measures such as accentedness rating and the role of syllable structure that interacts with acoustic, phonetic, and phonemic similarities between L1 and L2 speech sounds. The current data supplement these models by highlighting the role of syllable structure in learning L2 consonants. Further research efforts, including speech training, are needed to extend to the study of more complex syllable structure involving consonant clusters and the contribution of vowel contexts (e.g., Mok, 2010) and refine the multi-factor models as well as the underlying neural mechanisms that promote or limit neural plasticity in L2 acquisition (Greenberg, 2005; Zhang and Wang, 2007; Flege and MacKay, 2011). In addition to phonemic inventory, acoustic, and allophonic variations, suprasegmental phonotactic and phonological factors (including syllable-structure universals, co-articulation, assimilation, dissimilation, stress, and prosody), and the nature of linguistic input as shown in the recent development of corpus phonology (Durand et al., 2014) would all influence adult L2 learners’ perception and production. It is important to combine the segmental and suprasegmental approaches to understand how the matchup of phonetic/phonological units (including syllable structure) and allophonic variations between L1 and L2 shapes the perception and production in the course of L2 acquisition. The nature of L2 exposure experience, age of acquisition, language input, and attention would have to be incorporated in a successful model to account for and improve the perceptual and productive performance of L2 learners (Best and Tyler, 2007; Flege, 2007; Guion and Pederson, 2007; Aoyama et al., 2008; Flege and MacKay, 2011). As some Chinese dialects do not have some of the Mandarin phonemic distinctions (e.g., /n/ vs. /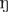/ and /n/ vs. /l/; Wang, 1991), it would also be interesting and important to study how dialectal background influences the positional asymmetry effect and the strength of perception-production links.

## Summary

The present study investigated the role of native language syllable structure in second-language phonetic perception and production. The overall scores show a strong positional asymmetry effect (i.e., better performance in syllable-initial position than in syllable-final position) on L2 consonantal perception and production across the categories of voicing, place of articulation, and manner of articulation. Moreover, a significant positive correlation between L2 perception and production was found in the syllable-initial position but not in the syllable-final position. There are also exceptions to the overall positional asymmetry pattern, suggesting that syllable structure is not the sole determinant. The results highlight the complexity of L2 speech learning and the necessity to consider syllable structure universals and native language interference by including syllable structure and allophonic variations in a multi-factor model for speech learning, which have important implications for future behavioral and brain research.

## Conflict of Interest Statement

The authors declare that the research was conducted in the absence of any commercial or financial relationships that could be construed as a potential conflict of interest.
