# Supplementary material for: Syllable Structure Universals and Native Language Interference in Second Language Perception and Production: Positional Asymmetry and Perceptual Links to Accentedness
Source: Front Psychol. 2015 Nov 26;6:1801. doi: 10.3389/fpsyg.2015.01801 (PMC4659920; doi:10.3389/fpsyg.2015.01801)
Supplement: Supplementary file 1 [file Supplementary_Material.PDF]

Supplementary Material

This supplemental document provides statistical results regarding potential dialectal influences that are referred to in the main text of the Frontiers in Psychology (Language Sciences Section) article (Cheng, B., & Zhang, Y. (2015). Syllable structure universals and native language interference in second language perception and production: Positional asymmetry and perceptual links to accentedness. Frontiers in Psychology, 6, 1801.).

Table S1 shows the multivariate analysis of variance (MANOVA) results for the perception data concerning the dialect factor for the between-subjects and within-subjects comparisons. The MANOVA tests treated dialect as a between-subjects main factor and the other main within-subjects factors include position (onset vs. coda) and sound category, which was run separately for voicing (voiced vs. voiceless), place of articulation (POA: bilabial, labiodental, interdental, alveolar, palatal-alveolar, and velar) and manner of articulation (MOA: nasal, plosive, fricative, affricate, and approximant). To simplify data reporting and interpretation, other main effects and interactions that do not involve the dialect factor are omitted from the table. None of the main factors or interactions involving dialect approached the level of statistical significance.

Table S2 shows the MANOVA results for the production data concerning the dialect factor for the between-subjects and within-subjects comparisons. To simplify data reporting and interpretation, other main effects and interactions that do not involve the dialect factor are omitted from the table. For place of articulation, there was one interaction effect (syllabic position * dialect) approaching significance [$F(1,36) = 3.717$, $p = 0.062$]. For manner of articulation, the between-subjects main factor of dialect showed a p value very close to the significance level [$F(1,36) = 4.104$, $p = 0.050$].

Table S3 shows the repeated measures ANOVA results for the perception data for all the major factors and interactions after the eight subjects with Wu or Min dialectal background were removed.

Table S4 shows the repeated measures ANOVA results for the production data for all the major factors and interactions after the eight subjects with Wu or Min dialectal background were removed.

Table S5 shows the Pearson's correlation test results at the onset and coda positions with eight subjects with Wu or Min dialectal background removed.

Table S1. MANOVA results for the perception data of the different sound categories. Only the main factor and interactions involving dialect are reported here.

**Sound Category**	**Perception**	
	**Between Subjects**	**Within Subjects**
Voicing	Dialect:<br>F(1,36) = 0.001, p = 0.974	Voicing*Dialect:<br>F(1,36) = 0.327, p = 0.571
		Position*Dialect:<br>F(1,36) = 0.126, p = 0.724
		Voicing*Position*Dialect:<br>F(1,36) = 0.282, p = 0.598
POA	Dialect:<br>F(1,36) = 0.003, p = 0.956	POA*Dialect:<br>F(5,180) = 0.935, p = 0.460
		Position*Dialect:<br>F(1,36) = 0.010, p = 0.920
		POA* Position*Dialect:<br>F(5,180) = 0.293, p = 0.858
MOA	Dialect:<br>F(1,36) = 0.378, p = 0.543	MOA*Dialect:<br>F(4,144) = 1.534, p = 0.218
		Position*Dialect:<br>F(1,36) = 0.007, p = 0.932
		MOA*Position*Dialect:<br>F(4,144) = 1.413, p = 0.247

Table S2. MANOVA results for the production data of the different sound categories. Highlighted in bold italics are the main and interaction effects approaching the significance level at 0.05. Only the main factor and interactions involving dialect are reported here. Greenhouse-Geisser correction is applied where appropriate.

**Sound Category**	**Production**	
	**Between Subjects**	**Within Subjects**
Voicing	Dialect:<br>F(1,36) = 0.568, p = 0.456	Voicing*Dialect:<br>F(1,36) = 1.92, p = 0.174
		Position*Dialect:<br>F(1,36) = 2.00, p = 0.166
		Voicing*Position*Dialect:<br>F(1,36) = 0.267, p = 0.609
POA	Dialect:<br>F(1,36) = 0.874, p = 0.357	POA*Dialect:<br>F(5,180) = 0.521, p = 0.675
		Position*Dialect:<br>***F(1,36) = 3.717, p = 0.062***
		POA* Position*Dialect:<br>F(5,180) = 1.832, p = 0.128
MOA	***Dialect:***<br>***F(1,36) = 4.104, p = 0.050***	MOA*Dialect:<br>F(4,144) = 0.645, p = 0.535
		Position*Dialect:<br>F(1,36) = 1.371, p = 0.249
		MOA*Position*Dialect:<br>F(4,144) = 0.399, p = 0.691

Table S3. Repeated measures ANOVA test and simple effects test results for the perception data from 30 subjects (eight subjects were removed due to concerns of dialectal influences). Significant effects are in bold italics. Greenhouse-Geisser correction is applied where appropriate.

**Sound Category**	**Perception**	
	**Main Factors and Interaction**	**Simple Main Effects**
Voicing	Voicing:<br>F(1,29) = 0.279, p = 0.601<br><br>***Position:***<br>***F(1,29) = 42.077, p < 0.00001***<br><br>***Voicing*Position:***<br>***F(1,29) = 4.973, p < 0.05***	***Position effect for voiced sounds:***<br>***F(1,29) = 4.541, p < 0.05***
		Position effect for voiceless sounds:<br>***F(1,29) = 24.843, p < 0.0001***
		Voicing effect at onset:<br>F(1,29) = 3.145, p = 0.087
		Voicing effect at coda:<br>F(1,29) = 3.108, p = 0.089
POA	***POA:***<br>***F(5,145) = 36.485, p < 0.00001***<br><br>***Position:***<br>***F(1,29) = 37.763, p < 0.00001***<br><br>***POA*Position:***<br>***F(5,145) = 5.753, p < 0.001***	***POA effect at onset:***<br>***F(5,145) = 33.249, p < 0.00001***
		POA effect at coda:<br>***F(5,145) = 16.164, p < 0.00001***
		Position effect for bilabials:<br>***F(1,29) = 11.410, p < 0.01***
		Position effect for labiodentals:<br>***F(1,29) = 11.528, p < 0.01***
		Position effect for interdentals:<br>F(1,29) = 0.013, p = 0.909
		Position effect for alveolars:<br>***F(1,29) = 135.097, p < 0.00001***
		Position effect for palatoalveolars:<br>F(1,29) = 2.027, p = 0.165
		Position effect for velars:<br>***F(1,29) = 5.178, p < 0.05***
MOA	***MOA:***<br>***F(5,145) = 34.370, p < 0.00001***<br><br>***Position:***<br>***F(1,29) = 155.075, p < 0.00001***<br><br>***MOA*Position:***<br>***F(5,145) = 41.850, p < 0.00001***	***MOA effect at onset:***<br>***F(4,116) = 15.727, p < 0.0001***
		MOA effect at coda:<br>***F(5,145) = 47.427, p < 0.00001***
		Position effect for nasals:<br>***F(1,29) = 29.487, p < 0.00001***
		Position effect for stops:<br>***F(1,29) = 47.764, p < 0.00001***
		Position effect for fricatives:<br>***F(1,29) = 6.351, p < 0.05***
		Position effect for affricates:<br>***F(1,29) = 43.875, p < 0.00001***
		Position effect for approximants:<br>***F(1,29) = 145.010, p < 0.00001***

Table S4. Repeated measures ANOVA test and simple main effects test results for the production data from 30 subjects (eight subjects were removed due to concerns of dialectal influences). Significant effects are in bold italics. Greenhouse-Geisser correction is applied where appropriate.

**Sound Category**	**Production**	
	**Main Factors and Interaction**	**Simple Main Effects**
Voicing	***Voicing:***<br>***F(1,29) = 107.977, p < 0.00001***<br><br>***Position:***<br>***F(1,29) = 23.256, p < 0.0001***<br><br>***Voicing*Position:***<br>***F(1,29) = 57.542, p < 0.00001***	***Position effect for voiced sounds:***<br>***F(1,29) = 45.9201, p < 0.000001***
		Position effect for voiceless sounds:<br>***F(1,29) = 6.672, p < 0.05***
		Voicing effect at onset:<br>F(1,29) = 3.464, p = 0.091
		Voicing effect at coda:<br>***F(1,29) = 106.789, p < 0.00001***
POA	***POA:***<br>***F(5,145) = 7.409, p < 0.001***<br><br>***Position:***<br>***F(1,29) = 19.430, p < 0.001***<br><br>***POA*Position:***<br>***F(5,145) = 11.212, p < 0.00001***	***POA effect at onset:***<br>***F(5,145) = 6.534, p < 0.001***
		POA effect at coda:<br>***F(5,145) = 9.941, p < 0.00001***
		Position effect for bilabials:<br>***F(1,29) = 13.470, p < 0.001***
		Position effect for labiodentals:<br>F(1,29) = 3.115, p = 0.087
		Position effect for interdentals:<br>***F(1,29) = 4.242, p < 0.05***
		Position effect for alveolars:<br>***F(1,29) = 35.017, p < 0.00001***
		Position effect for palatoalveolars:<br>F(1,29) = 0.192, p = 0.665
		Position effect for velars:<br>***F(1,29) = 34.160, p < 0.00001***
MOA	***MOA:***<br>***F(5,145) = 41.457, p < 0.00001***<br><br>***Position:***<br>***F(1,29) = 53.546, p < 0.00001***<br><br>***MOA*Position:***<br>***F(5,145) = 4.820, p < 0.05***	***MOA effect at onset:***<br>***F(4,116) = 26.979, p < 0.00001***
		MOA effect at coda:<br>***F(5,145) = 25.998, p < 0.00001***
		Position effect for nasals:<br>***F(1,29) = 14.459, p < 0.01***
		Position effect for stops:<br>***F(1,29) = 55.680, p < 0.00001***
		Position effect for fricatives:<br>F(1,29) = 0.254, p = 0.618
		Position effect for affricates:<br>F(1,29) = 0.632, p = 0.432
		Position effect for approximants:<br>***F(1,29) = 7.068, p < 0.05***

Table S5. Pearson's correlation analysis for the perception and production scores from 30 subjects in the onset and coda positions. Data from eight subjects were removed to address concerns of dialectal influences. Significant correlations are bold-underlined. (**p < 0.01; *p < 0.05.)

**Perception-Production Correlation**		**Onset**	**Coda**
**Overall**	Combined	**r = 0. 427, p = 0.018***	r = 0.199, p = 0.291
**Voicing**	Voiced	**r = 0.396, p = 0.030 ***	r = 0.204, p = 0.288
	Voiceless	**r = 0.481, p = 0.007 ****	r = 0.019, p = 0.923
**POA**	Bilabial	**r = 0.381, p = 0.038 ***	r = 0.106, p = 0.576
	Labiodental	r = 0.325, p = 0.080	r = 0.257, p = 0.170
	Interdental	r = 0.291, p = 0.119	r = -0.068, p = 0.723
	Alveolar	**r = 0.569, p = 0.001 ****	r = -0.208, p = 0.270
	Palatoalveolar	**r = 0.449, p = 0.013 ***	**r = 0.395, p = 0.031 ***
	Velar	**r = 0.364, p = 0.048 ***	r = 0.108, p = 0.570
**MOA**	Nasal	**r = 0.521, p = 0.003 ****	r = -0.101, p = 0.595
	Stop	**r = 0.445, p = 0.014 ***	r = 0.141, p = 0.458
	Fricative	**r = 0.461, p = 0.010 ***	r = 0.313, p = 0.093
	Affricate	r = 0.357, p = 0.053	r = 0.287, p = 0.124
	Approximant	**r = 0.477, p = 0.008***	r = 0.127, p = 0.504
